# Supplementary figures and images for: Adenovirus E1B-55K regulates p53-dependent and -independent gene expression during infection
Source: PLoS Pathog. 2025 Nov 3;21(11):e1013622. doi: 10.1371/journal.ppat.1013622 (PMC12591392; doi:10.1371/journal.ppat.1013622)

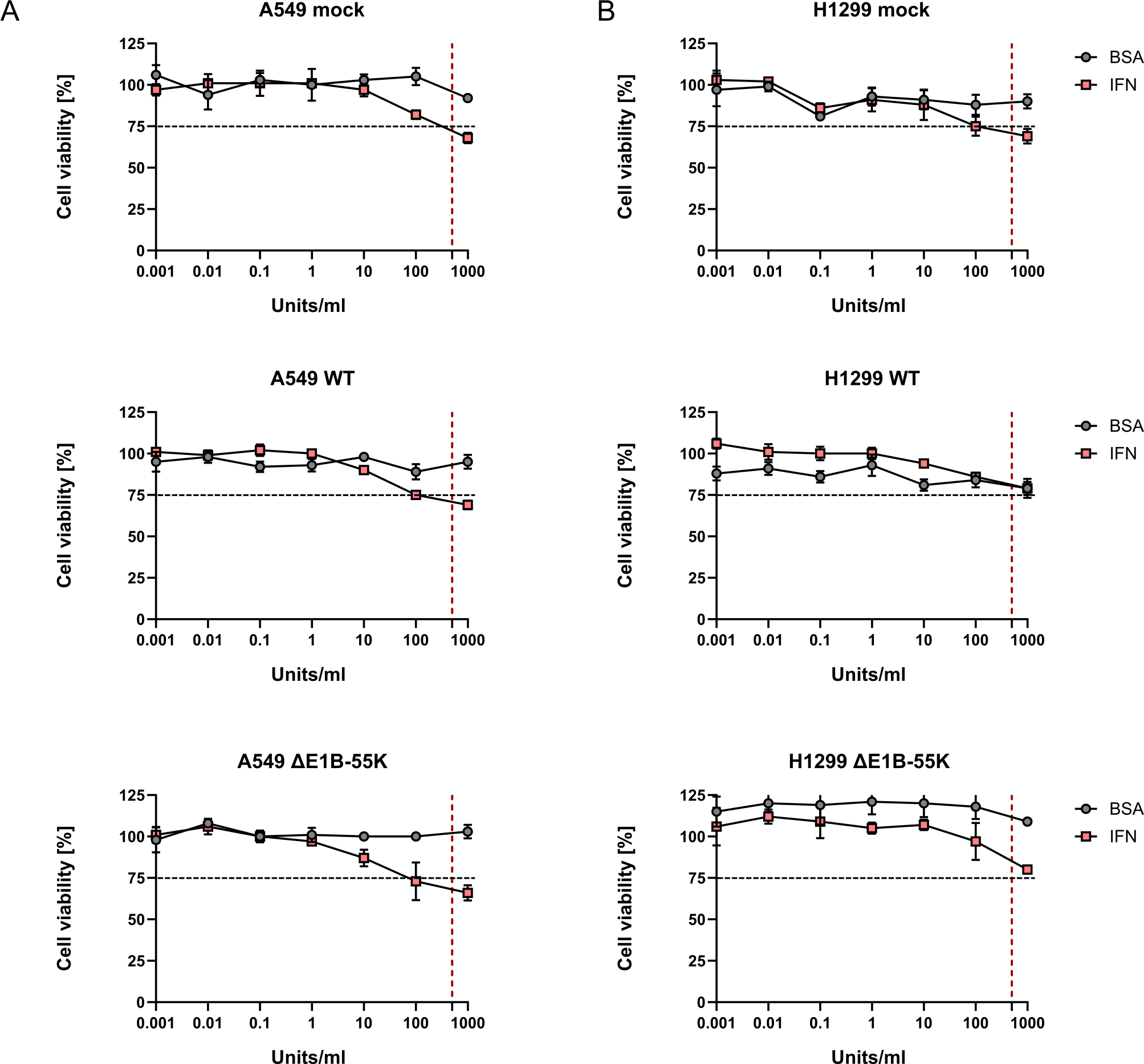

Supplement: S1 Fig — (A) A549 and (B) H1299 cells were treated with universal IFN-α or PBS supplemented with 0.1% BSA (carrier) at indicated concentrations for 16-18h. After pre-treatment cells were infected with MOI 30 of wildtype (WT) or ΔE1B-55K virus. At 24 hpi, cell viability was measured using the CellTiter-Glo Assay (Promega) on the Spark multimode microplate reader (Tecan). Data points represent the mean of three technical replicates. Error bars indicate SD. The red dotted line marks 500 U/ml, which was used in the other assays. The horizontal dotted line marks the 75% cell viability threshold. (TIF) [file ppat.1013622.s001.tif]

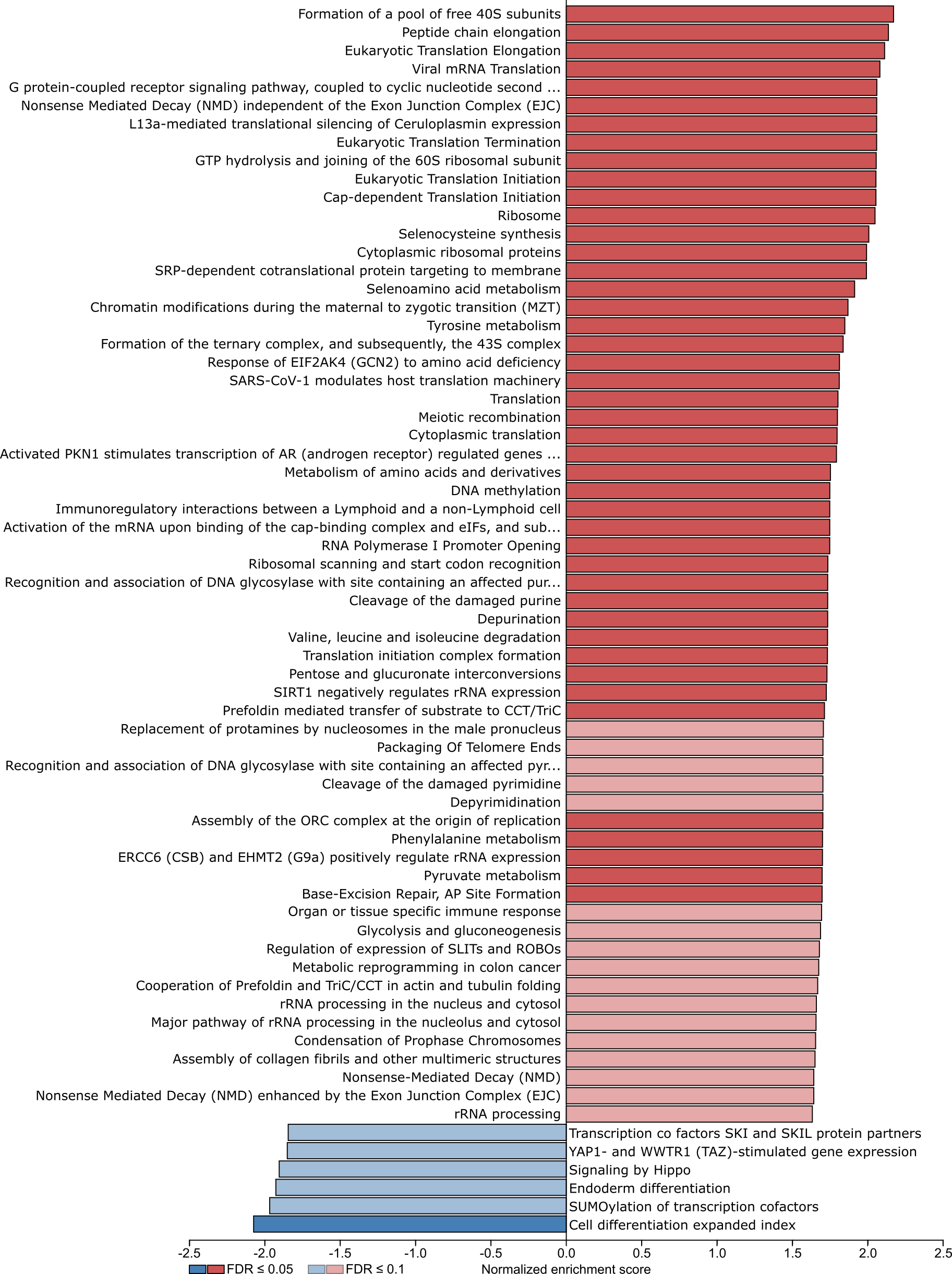

Supplement: S2 Fig — Wildtype and ΔE1B-55K infection at 24 hpi are compared in this analysis. Pathway analysis of up- and downregulated genes using GSEA, showing the normalized enrichment score of pathways with a FDR < 0.1 (light blue/red) and FDR < 0.05 (dark blue/red). Red bars indicate pathways that are enriched in upregulated genes, while blue bars indicate pathways that are enriched in downregulated genes. Databases utilized here were “Reactome”, “KEGG”, “WikiPathways” and “GO: Biological Process”. (TIF) [file ppat.1013622.s002.tif]

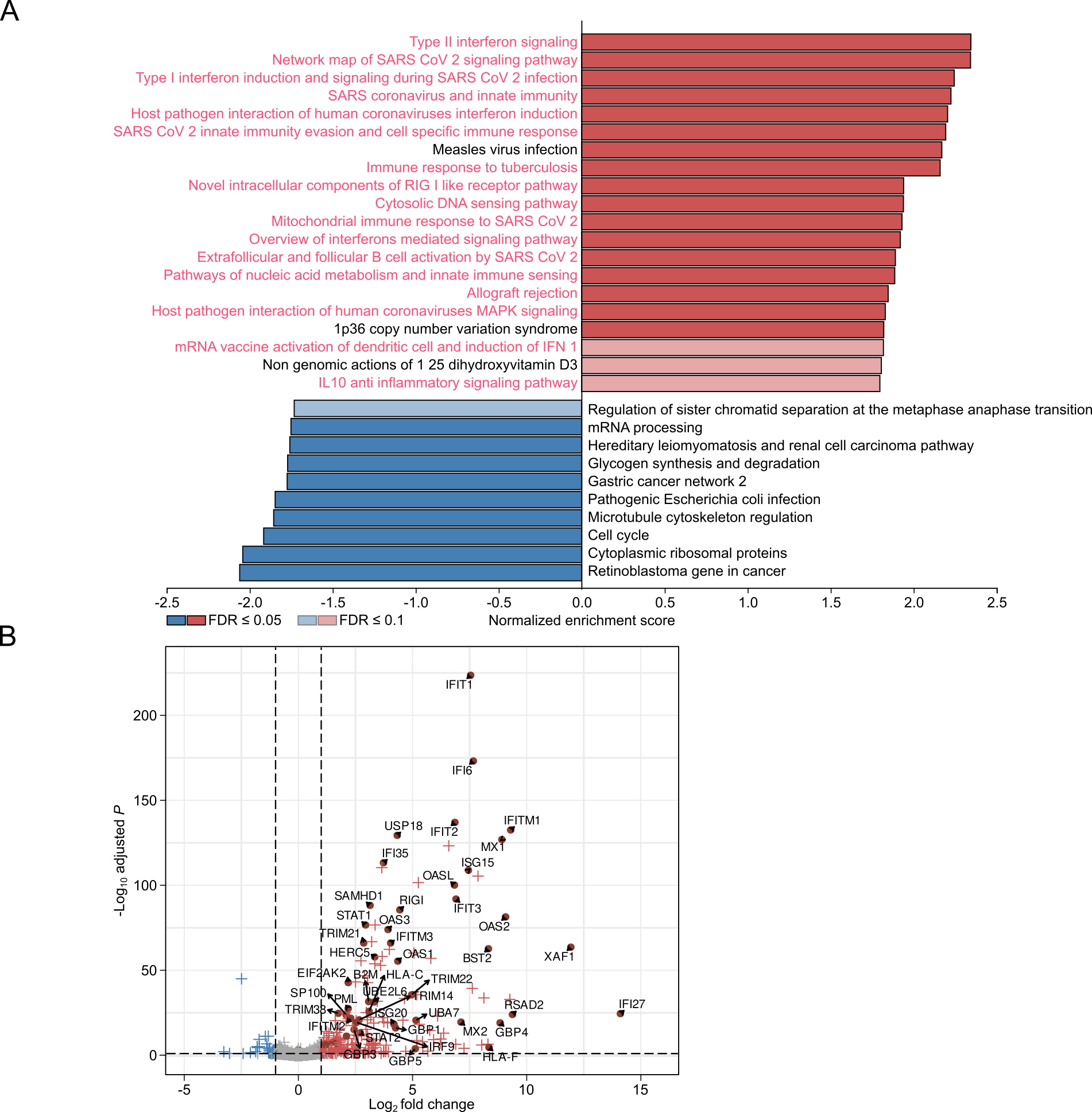

Supplement: S3 Fig — (A) Pathway analysis of up- and downregulated genes in mock-infected A549 cells comparing treatment with IFN-α or BSA using GSEA, showing the normalized enrichment score of pathways with a FDR < 0.1 (light blue/red) and FDR < 0.05 (dark blue/red). Pathways associated with the immune response are marked in pink. (B) Volcano plot of the comparison from (A). Genes with a padj. < 0.1 and log2 fold change < -1 were colored in blue, while genes with a padj. < 0.1 and log2 fold change > 1 were colored in red. Genes that belong to the “Interferon signaling” pathway from the Reactome database were additionally highlighted as dark red circles. (TIF) [file ppat.1013622.s003.tif]

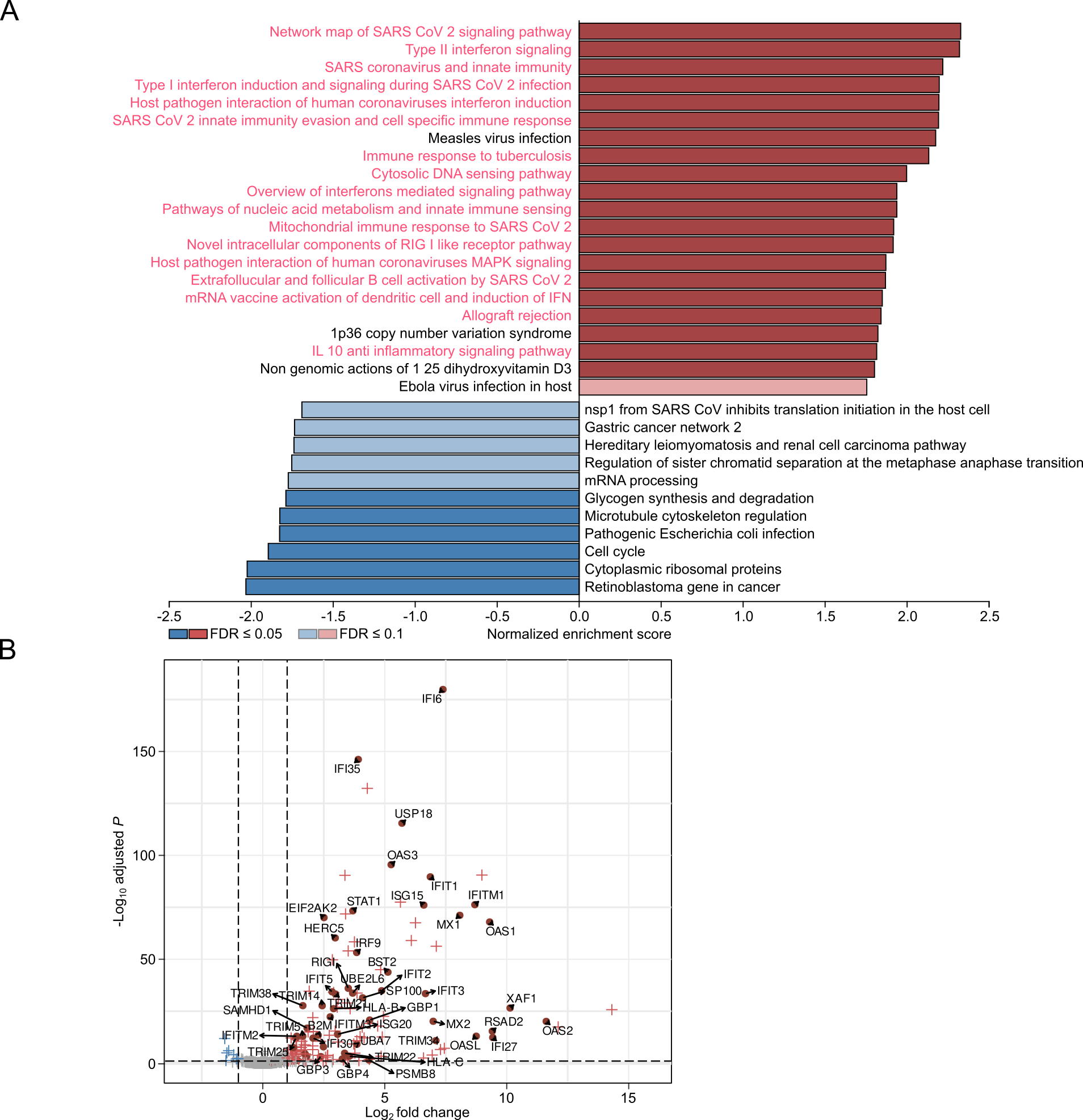

Supplement: S4 Fig — (A) Pathway analysis of up- and downregulated genes in mock-infected H1299 cells comparing treatment with IFN-α or BSA using GSEA, showing the normalized enrichment score of pathways with a FDR < 0.1 (light blue/red) and FDR < 0.05 (dark blue/red). Pathways associated with the immune response are marked in pink. (B) Volcano plot of the comparison from (A). Genes with a padj. < 0.1 and log2 fold change < -1 were colored in blue, while genes with a padj. < 0.1 and log2 fold change > 1 were colored in red. Genes that belong to the “Interferon signaling” pathway from the Reactome database were additionally highlighted as dark red circles. (TIF) [file ppat.1013622.s004.tif]

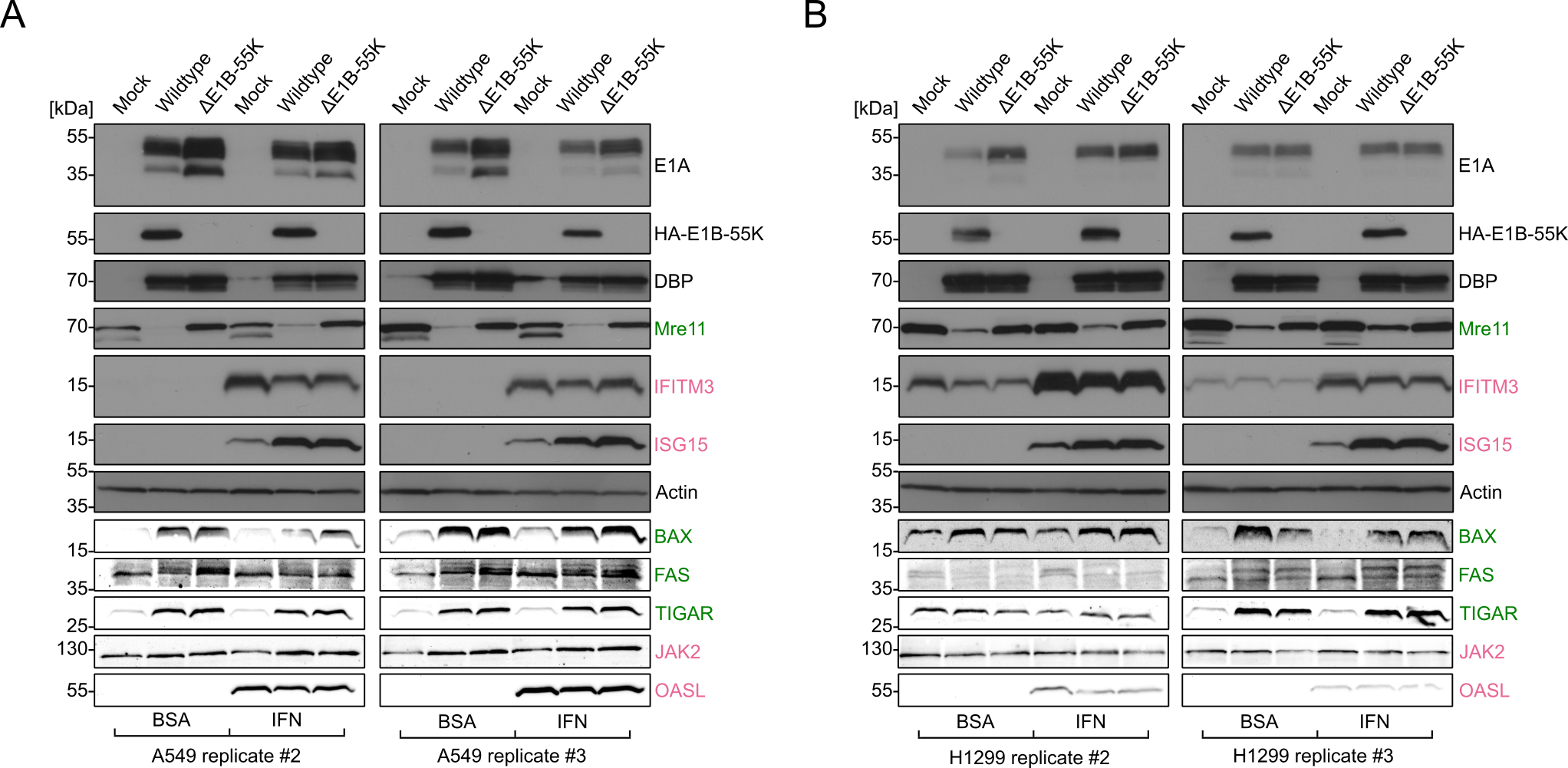

Supplement: S5 Fig — Western blot from sequenced replicates #2 and #3 of, displaying selected viral- and IFN-induced proteins as well as p53 target genes, as presented in Fig 3B. A549 (A) or H1299 (B) cells were treated with 500 U/ml IFN-α 16–18 h prior to infection with either wildtype or ΔE1B-55K virus. At 24 hpi, cells were harvested and RIPA extracts were prepared. For clarity, proteins associated with the p53 pathway are marked in green, while proteins associated with the immune response are marked in pink. The lower five proteins were visualized via the ChemoStar Plus, the upper ones were detected on medical X-ray films. Antibodies used for immunodetection can be found in S2 Table. (TIF) [file ppat.1013622.s005.tif]

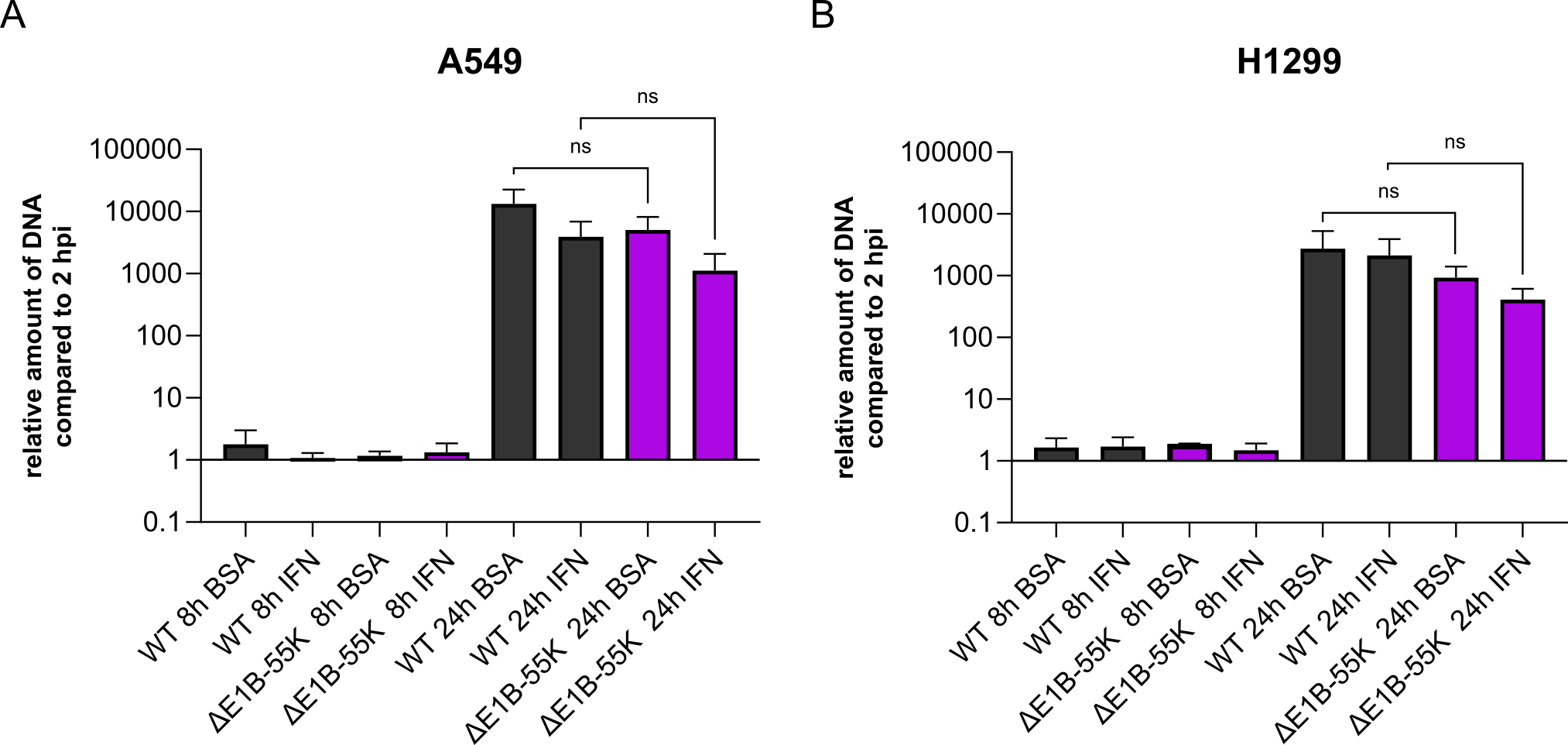

Supplement: S6 Fig — (A) A549 and (B) H1299 cells were treated with universal IFN-α (500 U/ml) or PBS supplemented with 0.1% BSA (carrier) for 16–18 h. After pretreatment cells were infected with MOI 30 of wildtype (WT) or ΔE1B-55K virus and harvested at 2, 8 and 24 hpi. Relative amount of viral DNA was determined by qPCR using a L4-100K-specific primer pair (S1 Table). Ct values were normalized to GAPDH. DNA concentrations are plotted relative to 2 hpi. Bar graphs represent the mean of three biological replicates, error bars indicate SD. Black and purple bars represent wildtype and ∆E1B-55K virus, respectively. Statistical significance was determined using a two-tailed t-test. Significance levels are indicated as follows: ns > 0.05. (TIF) [file ppat.1013622.s006.tif]

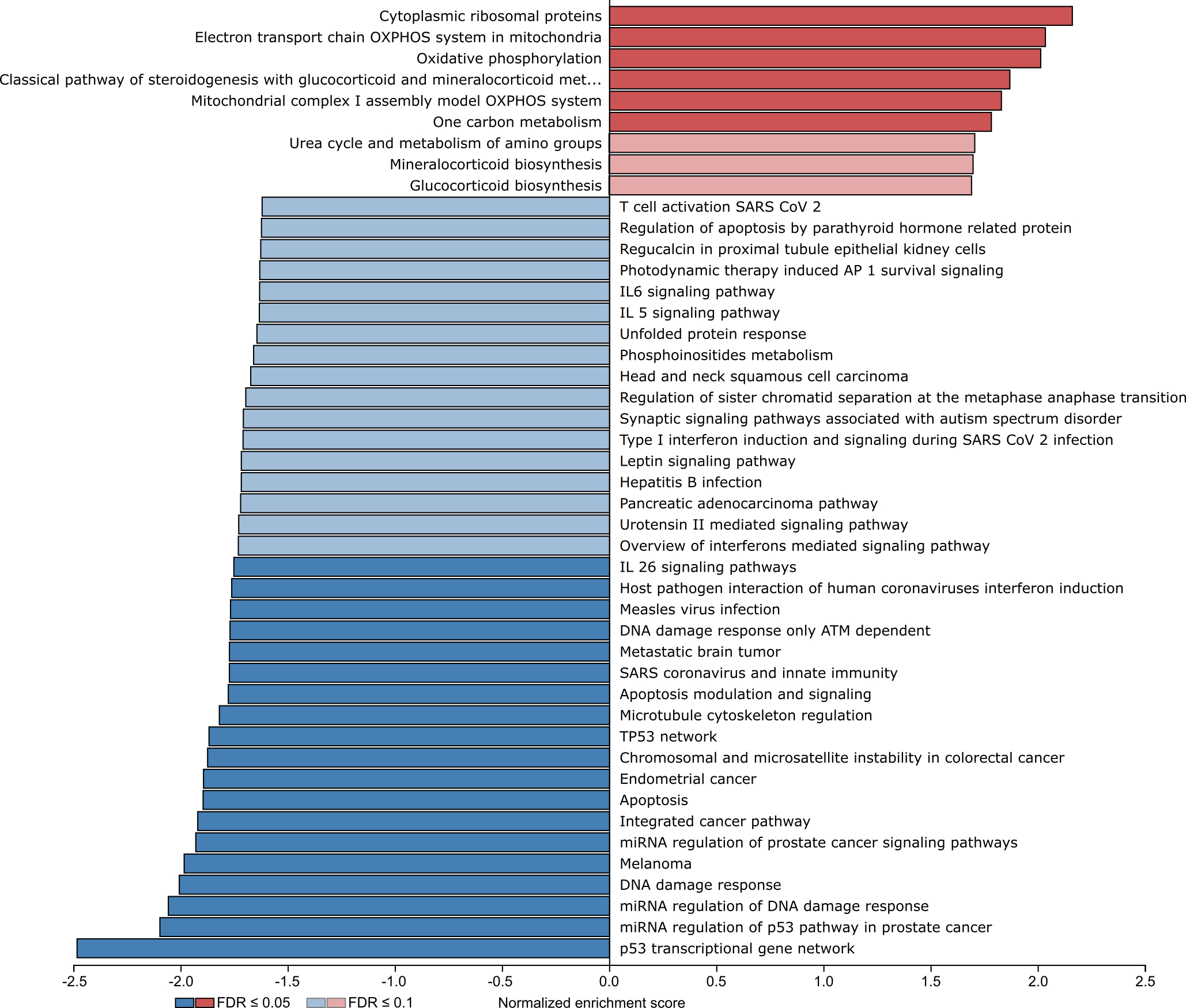

Supplement: S7 Fig — Wildtype and ΔE1B-55K infection at 24 hpi are compared in this analysis. Pathway analysis of up- and downregulated genes using GSEA, showing the normalized enrichment score of pathways with a FDR < 0.1 (light blue/red) and FDR < 0.05 (dark blue/red). The database utilized here is “WikiPathways”. (TIF) [file ppat.1013622.s007.tif]

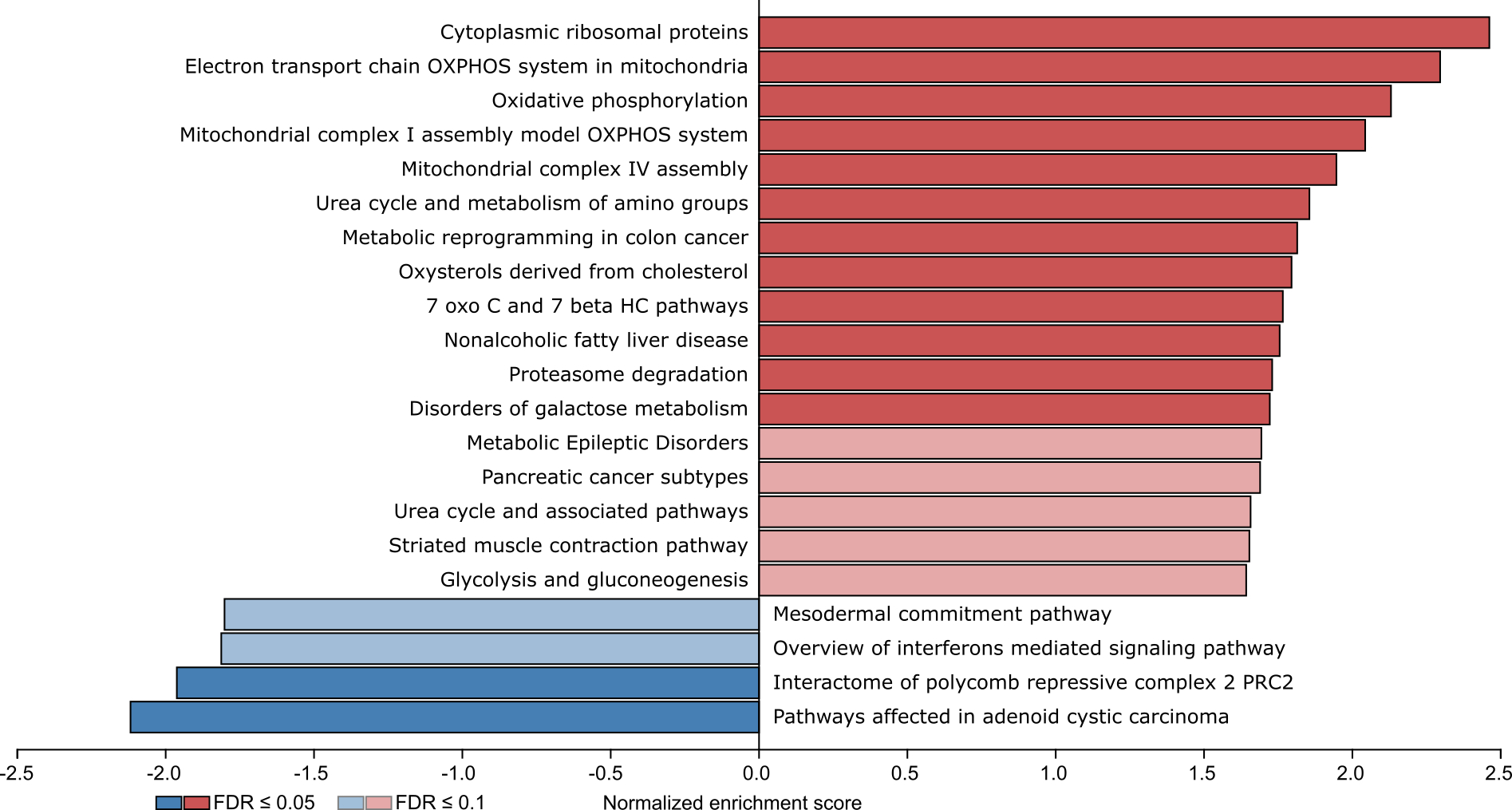

Supplement: S8 Fig — Wildtype and ΔE1B-55K infection at 24 hpi are compared in this analysis. Pathway analysis of up- and downregulated genes using GSEA, showing the normalized enrichment score of pathways with a FDR < 0.1 (light blue/red) and FDR < 0.05 (dark blue/red). The database utilized here is “WikiPathways”. (TIF) [file ppat.1013622.s008.tif]

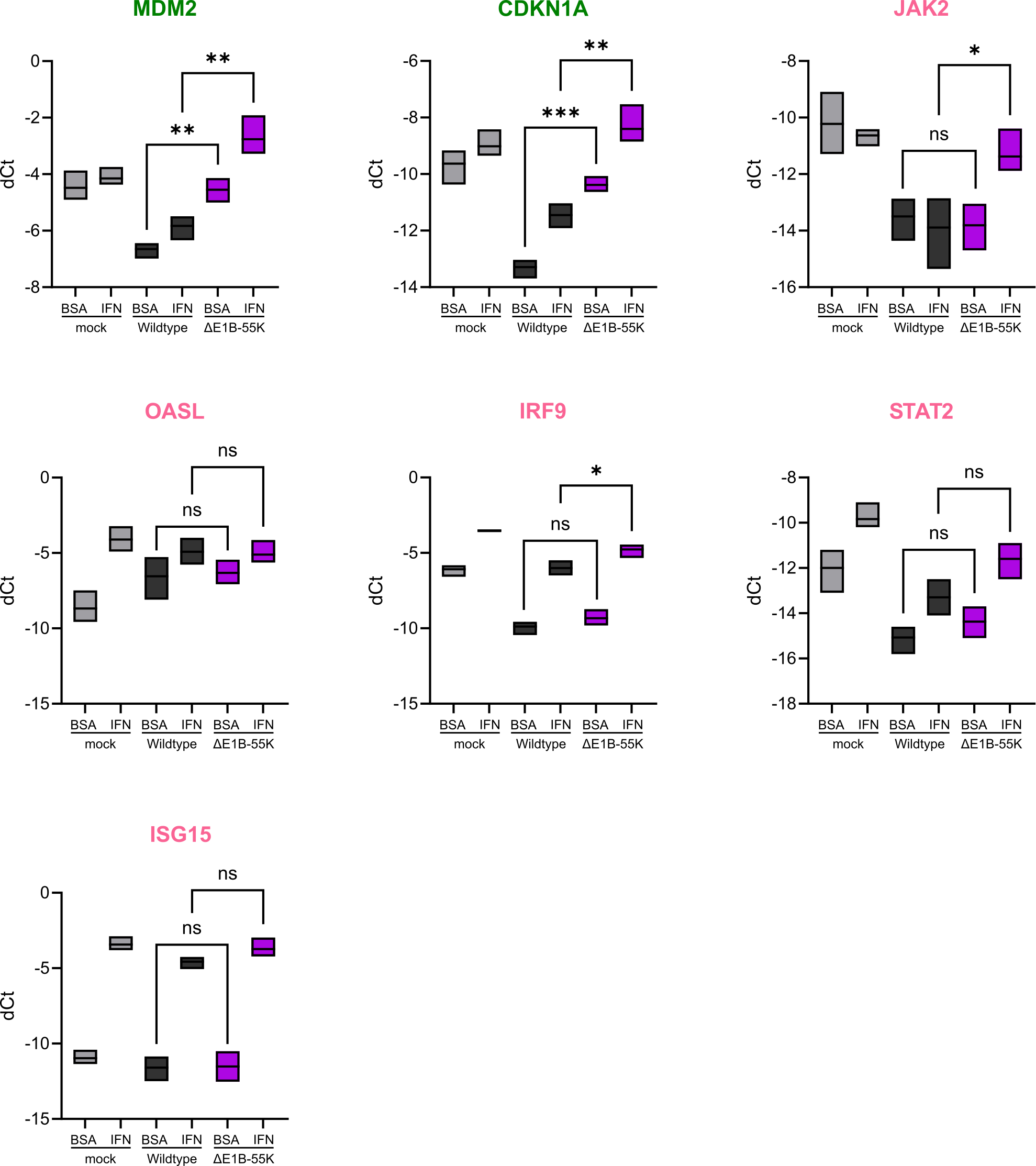

Supplement: S9 Fig — The GAPDH gene is an internal control used for normalization. Boxplots show means of dCt values (with min to max values) of the three independent biological replicates used in NGS. Light grey, black, and purple box plots represent mock, wildtype virus, and ΔE1B-55K virus conditions, respectively. Primers used in RT-qPCR can be found in S1 Table. Statistical significance was determined using a two-tailed t test. Significance levels are indicated as follows: * P-value < 0.05, ** P-value < 0.01, *** P-value < 0.001 and ns > 0.05. (TIF) [file ppat.1013622.s009.tif]

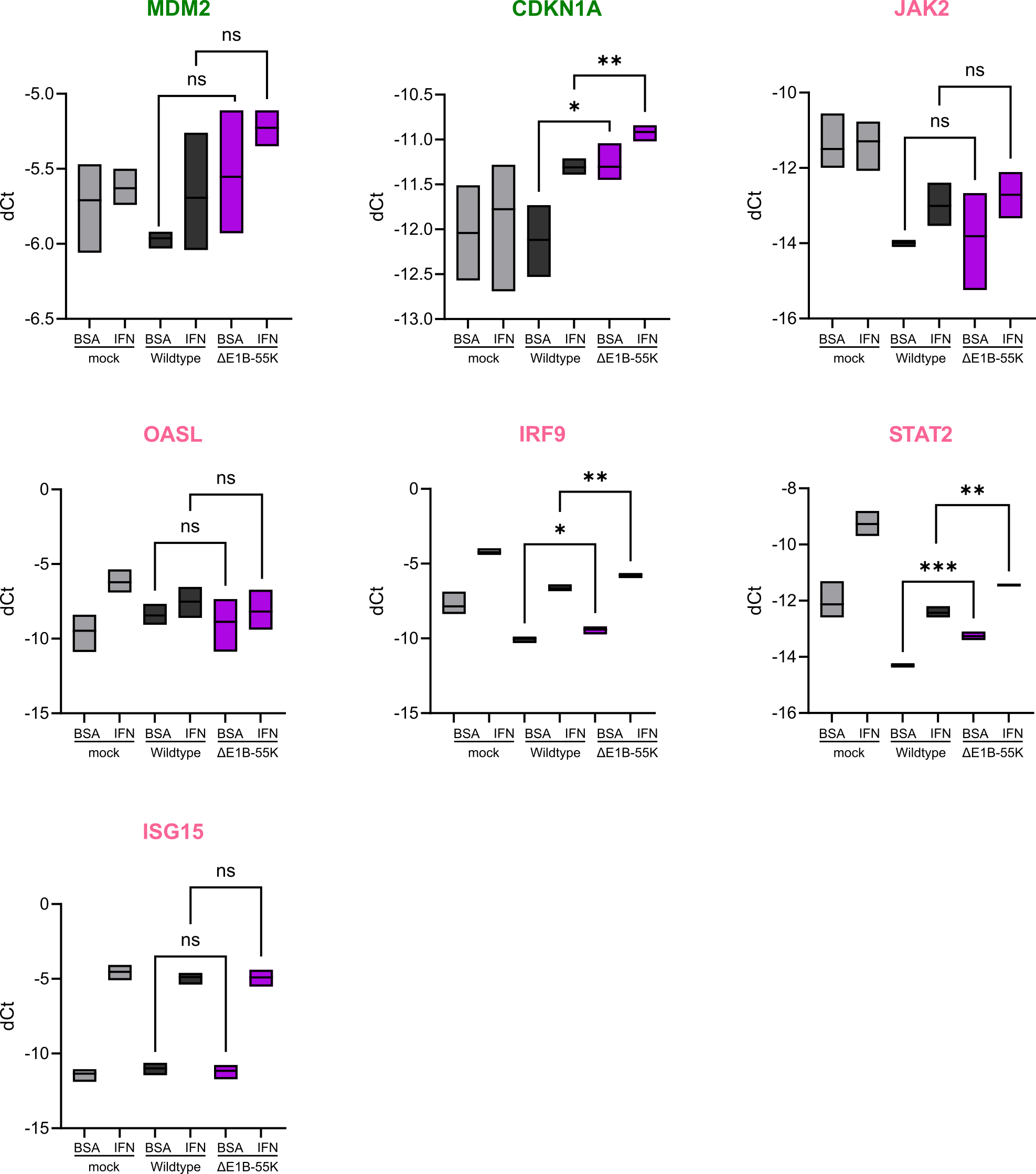

Supplement: S10 Fig — The GAPDH gene is an internal control used for normalization. Boxplots show means of dCt values (with min to max values) of the three independent biological replicates used in NGS. Light grey, black, and purple box plots represent mock, wildtype virus, and ΔE1B-55K virus conditions, respectively. Primers used in RT-qPCR can be found in S1 Table. Statistical significance was determined using a two-tailed t test. Significance levels are indicated as follows: * P-value < 0.05, ** P-value < 0.01, *** P-value < 0.001 and ns > 0.05. (TIF) [file ppat.1013622.s010.tif]

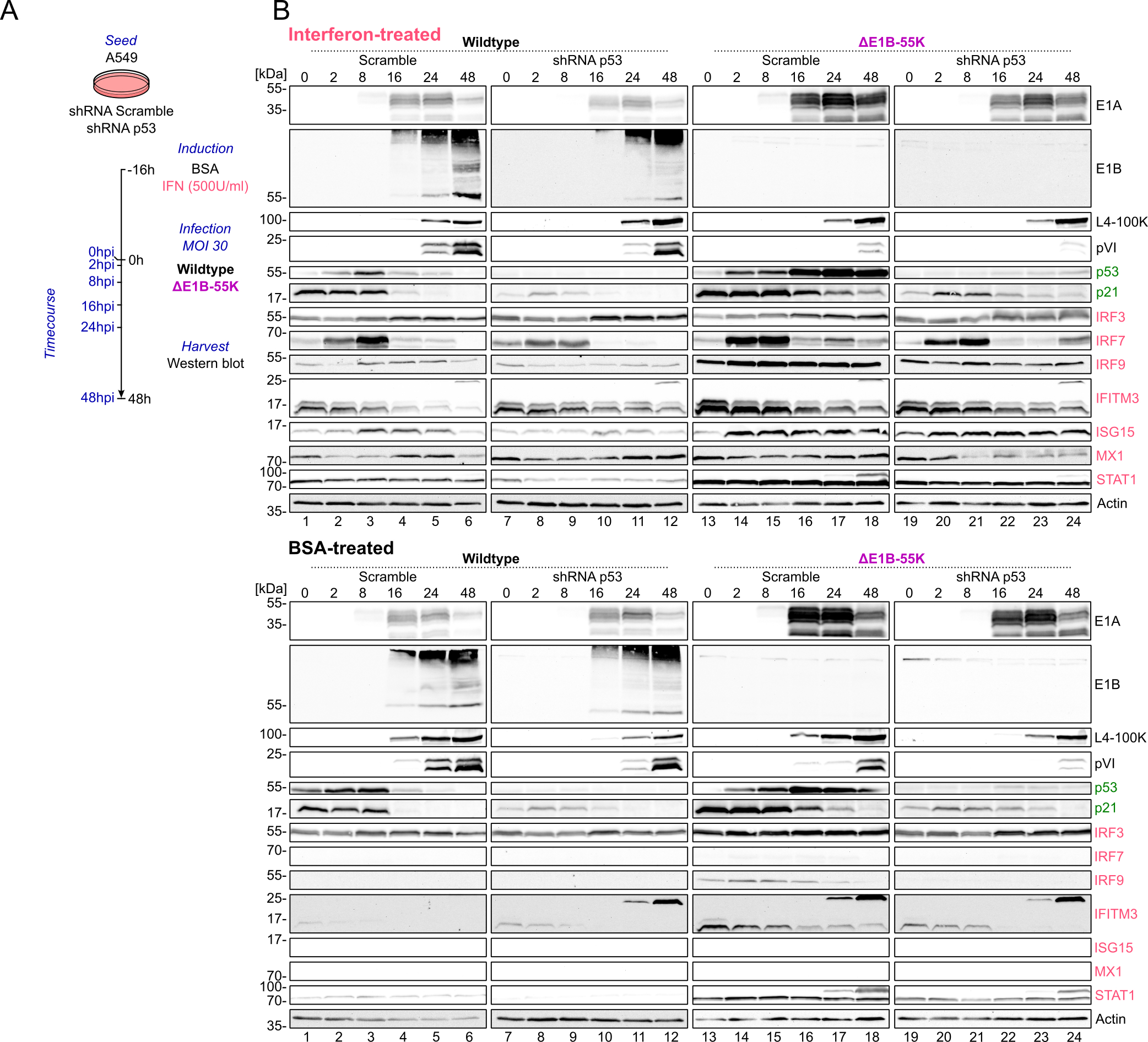

Supplement: S11 Fig — (A) IFN-α induction and time course scheme. (B) Western blot of IFN-treated (upper) or BSA-treated (lower) samples illustrating several adenovirus early and late proteins, as well as specific host cell p53- (green) and IFN-induced (pink) targets. Shown here is one replicate. The second replicate can be found in S12 Fig. Antibodies used for immunodetection can be found in S2 Table. (TIF) [file ppat.1013622.s011.tif]

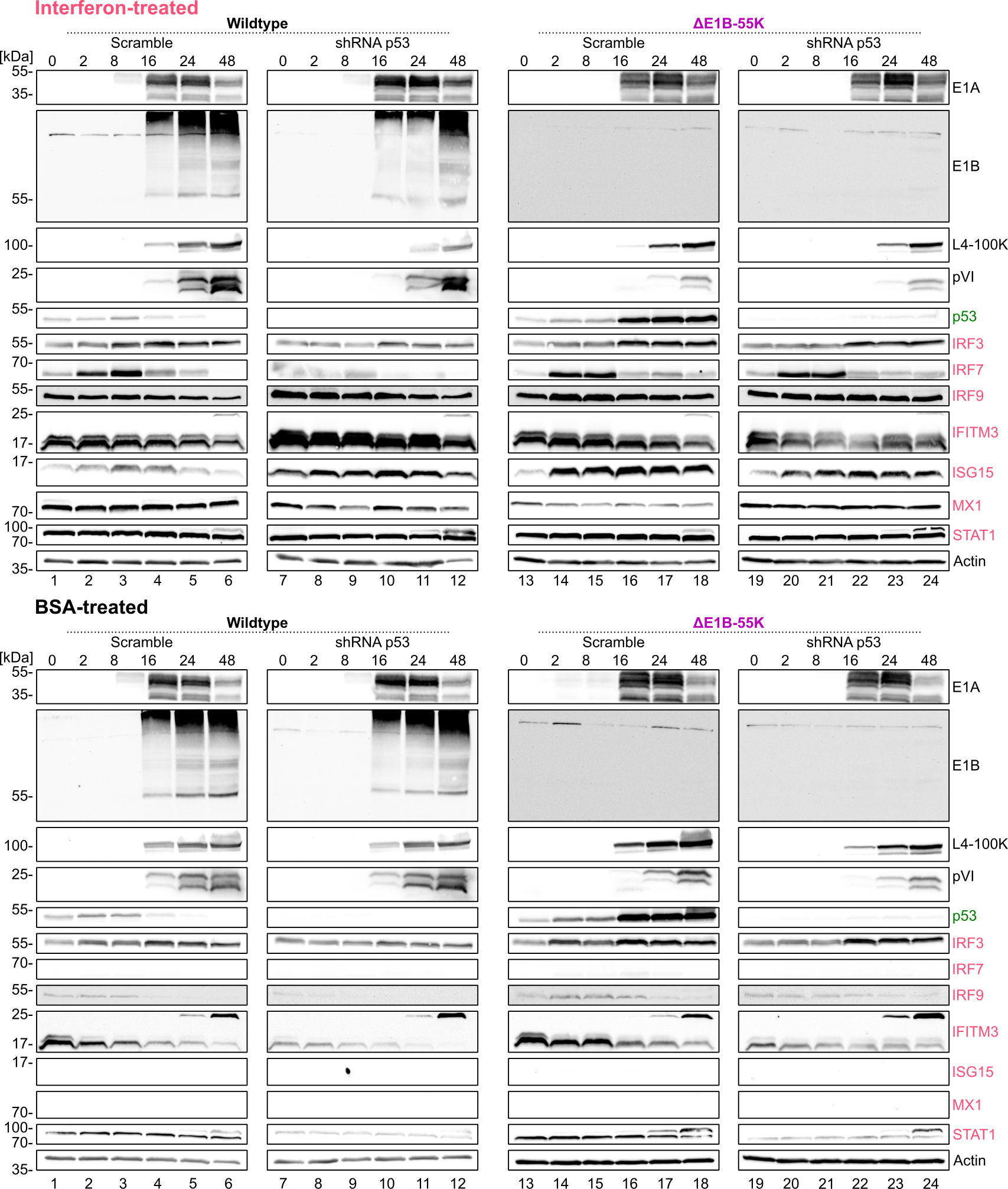

Supplement: S12 Fig — Western blot of IFN-treated (upper) or BSA-treated (lower) samples illustrating several adenovirus early and late proteins, as well as specific host cell p53- (green) and IFN-induced (pink) targets. Shown here is one replicate. The first replicate can be found in S11 Fig. Antibodies used for immunodetection can be found in S2 Table. (TIF) [file ppat.1013622.s012.tif]

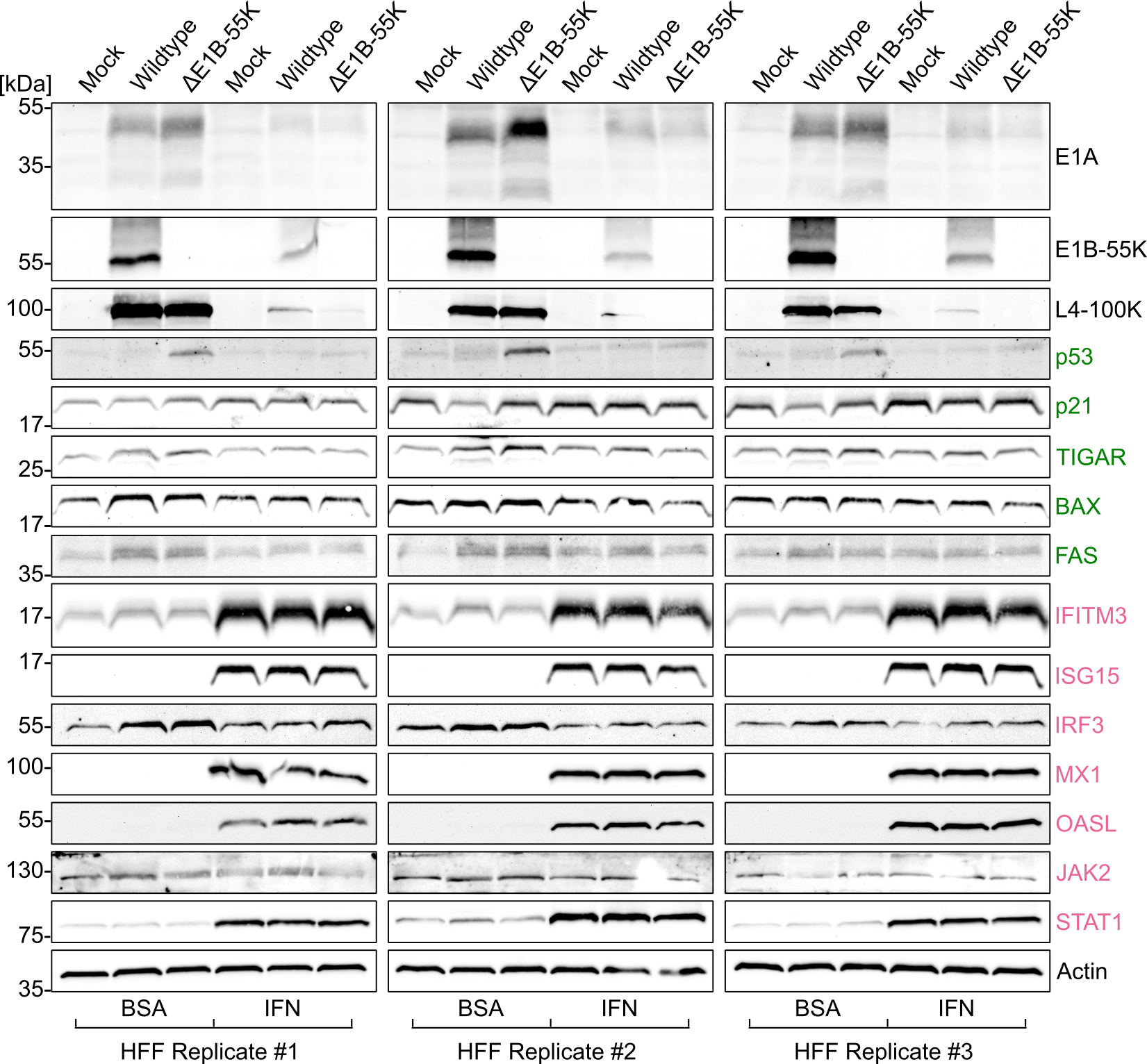

Supplement: S13 Fig — Western blot of three biological replicates, displaying selected viral-, p53-target (green) and IFN-induced (pink) proteins. HFF cells were treated with 500 U/ml IFN-α 16–18 h prior to infection with either wildtype or ΔE1B-55K virus. Cells were infected with MOI 100 and harvested at 48 hpi. Viral protein steady states are significantly reduced in the presence of IFN-α. Antibodies used for immunodetection can be found in S2 Table. (TIF) [file ppat.1013622.s013.tif]

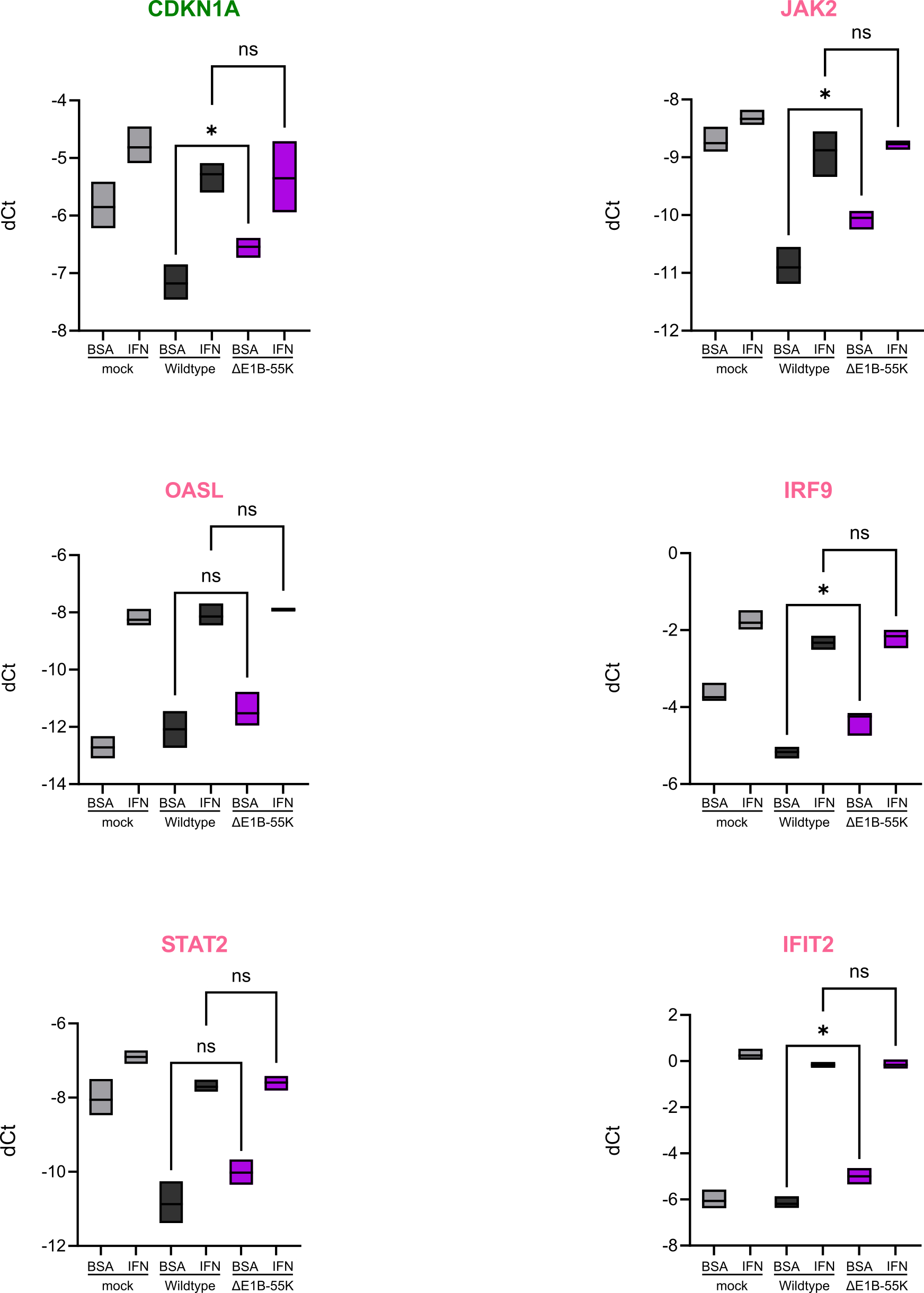

Supplement: S14 Fig — The GAPDH gene is an internal control used for normalization. Boxplots show means of dCt values (with min to max values) of the three independent biological replicates shown S13 Fig. Light grey, black, and purple box plots represent mock, wildtype virus, and ΔE1B-55K virus conditions, respectively. Primers used in RT-qPCR can be found in S1 Table. Statistical significance was determined using a two-tailed t-test. Significance levels are indicated as follows: * P-value < 0.05, and ns > 0.05. (TIF) [file ppat.1013622.s014.tif]
