# Supplementary material for: Adenovirus E1B-55K regulates p53-dependent and -independent gene expression during infection
Source: PLoS Pathog. 2025 Nov 3;21(11):e1013622. doi: 10.1371/journal.ppat.1013622 (PMC12591392; doi:10.1371/journal.ppat.1013622)
Supplement: S1 Table — (DOCX) [file ppat.1013622.s015.docx]

**S1 Table – Oligonucleotides**

| **Name** | **Purpose** | **Sequence (5‘ – 3‘)** |
| --- | --- | --- |
| HA-E1B-55K ccdB amp fwd | PCR | CCGGGGCGCGCTGCGGCTGCTGTTGCTTTTTTGAGTTTTATAAAGGATAAGCCAGTATACACTCCGCTAG |
| HA-E1B-55K ccdB amp rev | PCR | GGCCAGAAAATCCAGCAGGTACCCCCCGCTCAGATGGGTTTCTTCGCTCCAGCCCCATACGATATAAGTTG |
| HA-E1B-55K rescue | Rescue | CCGGGGCGCGCTGCGGCTGCTGTTGCTTTTTTGAGTTTTATAAAGGATAAATGTACCCATACGACGTCCCAGACTACGCTGAGCGAAGAAACCCATCTGAGCGGGGGGTACCTGCTGGATTTTCTGGCC |
| L4-100K fwd | RT-qPCR | AAACTAATGATGGCCGCAGTG |
| L4-100K rev | RT-qPCR | CGTCTGCCAGGTGTAGCATAG |
| GAPDH fwd | RT-qPCR | ACCACAGTCCATGCCATCAC |
| GAPDH rev | RT-qPCR | TCCACCACCCTGTTGCTGTA |
| IFIT2 fwd | RT-qPCR | GCGTGAAGAAGGTGAAGAGG |
| IFIT2 rev | RT-qPCR | GCAGGTAGGCATTGTTTGGT |
| IRF9 fwd | RT-qPCR | CAAGTGGAGAGTGGGCAGTT |
| IRF9 rev | RT-qPCR | ATGGCATCCTCTTCCTCCTT |
| STAT2 fwd | RT-qPCR | AATCGGCCTAGCAGTGCTTT |
| STAT2 rev | RT-qPCR | CCTCTAGCGCTGGCTTCTTT |
| JAK2 fwd | RT-qPCR | GCTGAAGCTCCTCTTCTTGATG |
| JAK2 rev | RT-qPCR | GCTGTGCAAGACAGAACTGC |
| OASL fwd | RT-qPCR | AAATTTCCAGGACCACCGCA |
| OASL rev | RT-qPCR | CTCTGGCTTCCTCAATCCCC |
| ISG15 fwd | RT-qPCR | CGCAGATCACCCAGAAGATT |
| ISG15 rev | RT-qPCR | GCCCTTGTTATTCCTCACCA |
| MDM2 fwd | RT-qPCR | gtgtatcaggcaggggagagt |
| MDM2 rev | RT-qPCR | CTTcaggaagccaattctcac |
| CDKN1A fwd | RT-qPCR | CTTGTAcccttgtgcctcgct |
| CDKN1A rev | RT-qPCR | cggattagggcttcctcttgg |
