# Supplementary material for: Adenovirus E1B-55K regulates p53-dependent and -independent gene expression during infection
Source: PLoS Pathog. 2025 Nov 3;21(11):e1013622. doi: 10.1371/journal.ppat.1013622 (PMC12591392; doi:10.1371/journal.ppat.1013622)
Supplement: S2 Table — (DOCX) [file ppat.1013622.s016.docx]

**S2 Table – Primary antibodies**

| **Antibody** | **Target** | **Species** | **Dilution** | **Source or reference** |
| --- | --- | --- | --- | --- |
| mAb HA tag (C29F4) | HA tag | Rabbit | 1:1000 | #3724; Cell Signaling |
| mAb M73 | E1A | Mouse | 1:10 | [1] |
| mAb 2A6 | E1B-55K | Mouse | 1:10 | [2] |
| mAb 6B10 | L4-100K | Rat | 1:10 | [3] |
| pAb pVI | pVI | Rabbit | 1:1000 | [4] |
| mAb B6-8 | DBP | Mouse | 1:10 | [5] |
| mAb AC-15 | β-actin | Mouse | 1:5000 | A-5441; Sigma-Aldrich |
| pAb FL-393 | p53 | Rabbit | 1:1000 | sc-6243 (discontinued); Santa Cruz |
| pAb MRE11 | MRE11 | Rabbit | 1:5000 | NB100-142; Novus |
| mAb TEF-3 (N-G2) | TEAD4 | Mouse | 1:500 | sc-101184; Santa Cruz |
| mAb WAF1/CIP1/CDKN1A p21 (F-5) | p21 | Mouse | 1:200 | sc-6246; Santa Cruz |
| pAb BAX | BAX | Rabbit | 1:1000 | #2772; Cell Signaling |
| mAb TIGAR (D3F4A) | TIGAR | Rabbit | 1:1000 | #14751; Cell Signaling |
| mAb FAS (C18C12) | FAS | Rabbit | 1:1000 | #4233; Cell Signaling |
| mAb JAK2 (D2E12) | JAK2 | Rabbit | 1:1000 | #3230; Cell Signaling |
| mAb OASL (E7W1R) | OASL | Rabbit | 1:1000 | #36845; Cell Signaling |
| mAb IFITM3 (D8E8G) | IFITM3 | Rabbit | 1:1000 | #59212; Cell Signaling |
| mAb ISG15 (F-9) | ISG15 | Mouse | 1:500 | sc-166755; Santa Cruz |
| mAb IRF-3 (D6I4C) | IRF-3 | Rabbit | 1:1000 | #11904; Cell Signaling |
| mAb IRF-7 (D2A1J) | IRF-7 | Rabbit | 1:1000 | #13014; Cell Signaling |
| mAb IRF-9 (D2T8M) | IRF-9 | Rabbit | 1:1000 | #76684; Cell Signaling |
| mAb MX1 (D3W7I) | MX1 | Rabbit | 1:1000 | #37849; Cell Signaling |
| mAb STAT1 (D1K9Y) | STAT1 | Rabbit | 1:1000 | #14994; Cell Signaling |

**References**

1. Debbas M, White E. Wild-type p53 mediates apoptosis by E1A, which is inhibited by E1B. Genes Dev. 1993;7(4):546-54. Epub 1993/04/01. doi: 10.1101/gad.7.4.546. PubMed PMID: 8384580.

2. Sarnow P, Sullivan CA, Levine AJ. A monoclonal antibody detecting the adenovirus type 5 E 1 b-58Kd tumor antigen: Characterization of the E 1 b-58Kd tumor antigen in adenovirus-infected and -transformed cells. Virology. 1982;120(2):510-7. doi: 10.1016/0042-6822(82)90054-x.

3. Kzhyshkowska J, Kremmer E, Hofmann M, Wolf H, Dobner T. Protein arginine methylation during lytic adenovirus infection. Biochem J. 2004;383(Pt 2):259-65. Epub 2004/07/10. doi: 10.1042/BJ20040210. PubMed PMID: 15242333; PubMed Central PMCID: PMCPMC1134066.

4. Wodrich H, Henaff D, Jammart B, Segura-Morales C, Seelmeir S, Coux O, et al. A capsid-encoded PPxY-motif facilitates adenovirus entry. PLoS Pathog. 2010;6(3):e1000808. Epub 20100319. doi: 10.1371/journal.ppat.1000808. PubMed PMID: 20333243; PubMed Central PMCID: PMCPMC2841620.

5. Reich NC, Sarnow P, Duprey E, Levine AJ. Monoclonal antibodies which recognize native and denatured forms of the adenovirus DNA-binding protein. Virology. 1983;128(2):480-4. doi: 10.1016/0042-6822(83)90274-x.
