# Supplementary material for: Adenovirus E1B-55K regulates p53-dependent and -independent gene expression during infection
Source: PLoS Pathog. 2025 Nov 3;21(11):e1013622. doi: 10.1371/journal.ppat.1013622 (PMC12591392; doi:10.1371/journal.ppat.1013622)
Supplement: Attachment — Submitted filename: Rebuttal Letter.docx [file ppat.1013622.s018.docx]

**Rebuttal Letter**

**Part I – Summary**

**Reviewer #1**: This short report examines the function of the human adenovirus type 5 E1B-55K protein in regulating host gene expression during the infection of two cell lines (A549 and H1299) using a mutant virus lacking E1B-55K and its parental strain. Transcriptomic and protein-based analyses confirm that E1B-55K suppresses p53-dependent transcription, a well-studied property which has primarily been investigated outside the context of adenovirus infection. Furthermore, E1B-55K was found to modulate interferon (IFN)-stimulated gene expression in A549 cells, but not in H1299 cells.

This study will be of great interest to the field. The results are clearly presented, and the conclusions are generally justified. However, the following issues need to be addressed.

Response: We appreciate the reviewer’s positive feedback and are pleased that our contributions have been accurately recognized.

**Reviewer #2**: The manuscript by Seddar et al studies the Adenovirus E1B-55K protein. This protein is best known as an oncogene that targets the p53 tumor suppressor. In addition, 55K also forms a complex with a second viral protein (E4orf6) and targets key cellular substrates for degradation including p53 and the Mre11 complex. In the current study, the authors show a previously unknown role for 55K in the regulation of interferon (IFN) responses.

The authors use transcriptomics approaches to study the differences in gene expression between wild-type Ad5 and mutant virus lacking 55K during infection of A549 and H1299 cells. As expected, the major differences are in the p53 pathway. What was less expected was that changes in IFN dependent gene expression were also observed. To follow up these observations, the authors go on to show that the E1B-55K null mutant is more sensitive to IFN pre-treatment. Since the p53 pathway is known to have cross talk with the IFN pathway, the conducted similar experiments in A549 cells in which p53 is knocked down. Under these conditions, IFN dependent gene expression is still suppressed suggesting that the mechanism is independent of p53.

Overall, the manuscript is an excellent contribution to our understanding of Ad E1B-55K and will be of general interest to DNA tumor virologists. The big remaining question is the mechanism by which 55K mediates its effects on IFN gene expression. Most likely, there is an unknown target of the orf6/55K complex that mediates these effects?

Response: We thank the reviewer for the positive feedback, which we believe accurately reflects the contributions of this paper.

**Reviewer #3**: Seddar et al. have performed a comprehensive RNA-seq based study of the role of E1B-55K during adenovirus infection, focusing on it’s role in controlling p53 signaling and in response to IFN treatment. The study is interesting and informative as previous papers tend to focus on E1B-55K’s role in transformation. The manuscript, however, suffer for some lack of details in many aspects and because of that can be more difficult to read and understand.

For example, the authors have generated RNA-seq data on many different sets of conditions, including mock, WT and 55K deleted infections; H1299 and A549 cell lines; control and IFN treated cells as well as some combinations. It is not always clear which comparison is shown, particularly in volcano plots and to which condition increases or decreases in reads refer to. In other cases, no description is provided in the text on how the experiments were conducted. Part of the issue may be due to space limitation for a short report. It might be worth considering a normal format to fully address the all the implication of the work.

Response: We thank the reviewer for their positive and constructive comments, and we are pleased that they found our study interesting and informative. We have now revised the figure legends and main text to explicitly indicate the conditions compared in each analysis.

**Part II – Major Issues: Key Experiments Required for Acceptance**

**Reviewer #1**: 1. The authors must validate any key differential changes identified in their RNA-seq analyses using RT-qPCR or a similar RNA-based method. Alternatively, they must justify why they believe such validation is unnecessary.

Response: We thank the reviewer for emphasizing the importance of validating our RNA-seq findings with an independent method. To address this, we selected representative p53-target genes and ISGs and performed RT-qPCR on the same samples used for the NGS data already presented in the manuscript. The results confirm the key differential changes and are now included in the supplementary data (Figs. S9 and 10).

**Reviewer #2**: No major issues.

**Reviewer #3**: No major issues

**Part III – Minor Issues: Editorial and Data Presentation Modifications**

**Reviewer #1**: 2. The authors should explain why they chose the A549 and H1299 cell lines for this study. Cancer cell lines often exhibit altered IFN and p53 responses. Apart from the fact that the latter are p53-negative, what is known about the IFN and p53 responses in A549 and H1299 cells? The data should be discussed in this context.

Response: We thank the reviewer for the opportunity to clarify our rationale regarding our choice of cell lines. We fully agree that the altered p53 and IFN responses in A549 and H1299 cell lines are critical for interpreting our data. To address this, we have expanded the discussion to outline both the advantages and the limitations of using these models in our study. Our rationale was guided by previous work from Chahal et al., which suggested that E1B-55K interferes with IFN signaling. Based on this, we hypothesized that such deregulation might occur via transcriptional repression of p53, given the substantial evidence in the literature supporting cross-talk between p53 and IFN signaling (lines 312-318). Moreover, we show that both cell lines show robust expression of a multitude of ISGs after induction with interferon (Fig. S3 and S4). Lastly, numerous studies investigating influenza and other RNA viruses employ A549 cells as a model system to elucidate their interactions with host interferon responses (See, e.g., 10.1016/j.jviromet.2023.114731, 10.1016/j.vetmic.2022.109390, 10.1016/j.molcel.2023.11.032 and others).

3. An imbalance exists between the data presented in the main manuscript (3 figures) and that included in the supplement (2 tables and 11 figures, as well as additional methods). It is unclear why the authors decided to present their work as a short report rather than a full-length one, which would have allowed more figures to be included in the main manuscript.

Response: Thanks. We chose to present the supplementary information separately because the data included there are indeed supplementary and support the main findings without disrupting the flow of the manuscript. However, in response to your comment and since we are now resubmitting the manuscript as a full-length paper rather than a short report, we have decided to include Fig. S2 in the main manuscript to better balance the presentation of key data.

4. Figure 1A: The text should mention that reduced levels of Mre11, p53 and CDKN1A were not observed in the E1B-55K mutant virus.

Response: Included (lines 199-201).

5. Figure 1D: To say that 'these genes remained transcriptionally active in both mock-infected and ΔE1B-55K-infected cells' is an overstatement, since many p53-responsive genes are downregulated in cells infected with either the wild-type or mutant viruses, albeit to different extents.

Response: Thanks! We revised the text accordingly (lines 205-207).

6. The regulation of pathways other than those dependent on p53 and IFN by E1B-55K should be discussed, including its potential effects on metabolism, as illustrated in Figure 1E.

Response: Thank you for this very valuable comment. Since we are now resubmitting the manuscript as a full-length paper rather than a short report, we have expanded the discussion to address additional pathways deregulated by E1B-55K during infection, including those beyond p53- and IFN-dependent regulation, as illustrated in Figure 1E (lines 214-217 and 364-376).

7. The conclusions that 'infection with adenovirus also affected the TEAD transcriptional network' (line 143) and 'our analysis revealed consistent downregulation of the Hippo signaling pathway' (line 144) appear to apply to infected H1299 cells, but not to A549 cells. This should be clearly stated and discussed in the text. Additionally, the text should specify which TEAD protein (TEAD4) was investigated for physical interaction with E1B-55K in Figure S2.

Response: We adapted the results section accordingly.

8. The authors should acknowledge that the IFN-related differences they observe between wild-type and mutant viruses, as well as between A549 and H1299 cells (Figures 2 and 3), tend to be small. It is debatable whether some of these effects are genuine or merely spurious (e.g. differences in viral DNA replication between A549 and H1299 cells in Figure 2C).

Response: Thank you for this constructive feedback. The reviewer is right, this is why we have already described the effect as ‘mild’ (line 262), also specified that ‘E1B-55K moderately contributes to the suppression of ISG expression…’ (lines 85-86)” and emphasize that we observed “some individual genes to be significantly repressed in A549 but not H1299 cells, albeit only on a transcriptional and not protein level” (lines 270-273). In addition, we have further refined our descriptive language when presenting the transcriptomic data to better reflect the modest nature of these effects. Of note, repression of specific ISGs was confirmed by RT-qPCR (Fig. S9 and S10).

9. Although the manuscript is written well overall, it should be carefully checked for small spelling errors and inconsistencies. These include, but are not limited to, 'by targeting *a* host tumour suppressor' (Abstract) and 'displaying *raw* Z-scores' (Figure 1D legend).

Response: Thanks, we checked the whole manuscript and SI again for spelling errors and inconsistencies.

10. The text shows signs of potential AI (LLM) use. If the authors have used AI in the writing process, they should acknowledge this.

Response: We confirm that no LLM or AI-based tool was used to generate the text of the manuscript. All writing was carried out by the authors.

**Reviewer #2**: The manuscript is very well written and referenced. The data is nicely presented with all relevant statistics provided. The only suggestion I can make for improvement is that the molecular weight markers in supplemental figures 8 and 9 are missing. The authors should include these as they have with the other immunoblots in the manuscript.

Response: Included, thanks!

**Reviewer #3**:Detailed issues

- In the results section, the description of Figure 1 starts very abruptly without details of the experiment. The first sentence starts with: “Western blot analysis confirmed productive infection …”. Also the data in fig. 1A does not actually confirm a productive infection as it is only showing expression of 55K, some substrates of the orf6/55K ligase complex, and down stream targets of p53. The wording should be modified to be more precise.

Response: Thanks, we adapted the manuscript accordingly (lines 193-195).

- Fig. 1E are red (increased) lines indicate increased in mutant infected cells relative to the wt infected cells? This should be clearly indicated.

Response: Agreed, we added information to the figure legend to clarify the gene set enrichment analysis (GSEA).

- Line #143-147. No details of what the experiment was or cell line. The text suggests WT infection compared to no infection, however, the Figure legend for S2 seems to indicate WT infection compared to mutant virus infection. Should be clarified.

Response: Thanks, we included the details as requested.

- Fig. S3 “Pathway analysis of up- and downregulated genes” Need to precise what is being compared here.

Response: We have revised the figure legend to specify the comparison underlying the GSEA.

- Line #172-173 The wording in incorrect. “IFN-α exacerbated the replication defect of the ΔE1B-55K mutant, particularly in H1299 cells (Figs. 2C and 3A).” In 2C it only shows that there is a replication defect in A549 cells while panel 3A does not show any replication data. Need to be changed.

Response: Changed as per reviewer suggestion.

- Which data set is shown in Fig 3B? it is not clear.

Response: Thanks, we added the requested information to the figure legend.

More minor issues and typos
- Fig. 1C The text says that p53 target genes are marked in green. At leas on my screen all dots look black

Response: We have carefully checked the figure on multiple devices and displays, and in our version, all p53-targets are indeed marked in green.

- Fig. 1D The grey, black and purple boxes shown on the top of the panel probably indicate the same as in panel B, but that should be noted in the figure legend.

Response: Done.

- Supplementary Figure 1 is not mentioned in the text. It should be or otherwise removed.

Response: Fig. S1 is referenced in the M&M paragraph “Viruses and infections” (line 109).

- Line #143 “infection with adenovirus also affected the TEAD transcriptional network” SF2 should be indicated here

Response: Good point, thanks!

- Fig S2 legend title mentions in absence of p53, but the cell line used, H1299 should be mentioned at the beginning, not just for panel E. The letters for panel C, D and E should be in bold like A and B.

Response: Thanks, we corrected the legend title and the font.

- Fig S2C refers to Fig. S2. That should be Fig. S3

Response: Corrected.

- Figure legend for Fig. S2E. “expression of transfected E1B-55K and exogenous TEAD4” What does exogenous means here? It appears to also be transfected.

Response: We clarified the misleading statement in the figure legend.

- Fig. 2D should specify in the legend what the white and pink banding means. It’s on the figure, but not obvious and hard to see.

Response: We have updated the legends of Figs. 2 and 3 to clarify that the pink banding indicates IFN treatment, while the white banding represents the control condition.

- Line #160. Should be Fig 2B and not 3B

Response: Yes, thanks for spotting this mistake!

- Line #166 the 24 post infection should be 24h

Response: Corrected.

- SF7 legend. Only need to describe the 1 star as there is only that condition in the figure

Response: Corrected.

- Line #178 “ISG expression was consistently lower in infected cells than in the uninfected” That is not the case for all.

Response: Thanks for pointing that out, we corrected the sentence (lines 266).

- Figure legend for panel 3D need to indicate which cell line is on the left and right.

Response: Included, thanks!
